# When Right Isn’t Right: Left Femoral Access for EVOQUE Transcatheter Tricuspid Valve Replacement System

**DOI:** 10.1016/j.shj.2025.100724

**Published:** 2025-08-13

**Authors:** Antonio H. Frangieh, Mark W. Abdelnour, Siddharth Vad, Scott Chadderdon, Jin Kyung Kim, Firas Zahr

**Affiliations:** a Mary & Steve Wen Cardiovascular Division, University of California Irvine, Irvine, California, USA; b Transcatheter Mitral and Tricuspid Therapies Division, Edwards Lifesciences Corporation, Irvine, California, USA; c Division of Cardiovascular Medicine, Oregon Health & Science University, Portland, Oregon, USA

**Keywords:** EVOQUE, IVC-TVA offset, Left femoral access, RA height, Transcatheter tricuspid interventions, Transcatheter tricuspid valve replacement, TTVR

## Abstract

As transcatheter tricuspid valve replacement with the EVOQUE system gains wider clinical adoption, growing experience has highlighted key anatomical considerations that influence procedural success. While right transfemoral (TF) access is the standard approach, it can be technically challenging in patients with complex right heart anatomy, such as low right atrium (RA) height or a large inferior vena cava–tricuspid valve annulus (IVC-TVA) offset. These factors may lead to suboptimal trajectory, impaired coaxiality, and difficult valve deployment. Left TF access offers a potential alternative by providing additional RA height and a more favorable lateral trajectory, allowing improved alignment with the tricuspid valve annulus (TVA). This approach is particularly useful in patients with large short-axis (SAX) offsets (>20 mm) or steep long-axis angles where right TF access may not achieve perpendicular orientation despite secondary catheter flexion. Using a preprocedural cardiac computed tomography angiography (CCTA) scan, anatomical factors such as RAH, leaflet tethering height, RV depth, and papillary muscle location can be evaluated to guide access planning. While left TF access introduces its own technical considerations, including venous tortuosity, excessive unwanted RA height, and increased need for primary flex, it may expand procedural feasibility in anatomically challenging cases. This review outlines real-world scenarios where left-sided access was favored, supporting its use as a safe and effective strategy in selected patients. Further studies are warranted to assess long-term outcomes and to inform the design of next-generation delivery systems capable of accommodating broader anatomical variation.

## Highlights

• The left TF approach in EVOQUE TTVR can be considered in specific challenging right heart anatomies.
• In specific right heart anatomies, such as low RA height (<60 mm) or large IVC–TVA offset (>20 mm), the left TF approach offers added RA height and a more favorable lateral trajectory for better TVA alignment.
• Preprocedural CT can help assess anatomical factors like RA height, IVC-TVA offset, leaflet tethering height, RV depth.

## Graphical abstract

**Figure:**
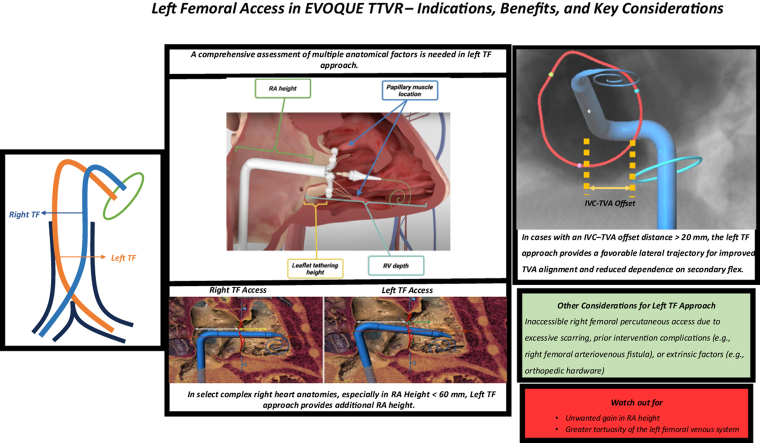

As transcatheter tricuspid interventions continue to evolve in design, indications, and approvals, growing clinical experience offers valuable procedural tips and tricks that can help address both anatomical challenges and device limitations. Transcatheter tricuspid valve replacement (TTVR) systems vary significantly in valve design, stent frame, anchoring mechanisms, valve sizing, and delivery system characteristics, including French size, access route, number of catheters, and deployment mechanisms.^1^ The EVOQUE system (Edwards Lifesciences, Irvine CA) is the first dedicated TTVR device to receive both the CE (Conformité Européene/European Conformity) mark and US Food and Drug Administration approval and is being increasingly utilized for the treatment of symptomatic severe tricuspid regurgitation. The EVOQUE system is designed for transfemoral (TF) access, although transjugular (TJ) access has been recently suggested to be utilized in particular challenging anatomies, as it can provide a shorter and less tortuous path to the right atrium (RA), with more favorable coaxial alignment between the superior vena cava and the center of the tricuspid valve annulus (TVA).^2^ However, TJ access may present challenges due to large internal jugular (IJ) bore size access, operator unfamiliarity with catheter manipulation from the neck and more radiation exposure.^1^ In contrast, TF access, particularly from the right side, is often favored due to its routine use in most transcatheter structural heart procedures. In the TRISCEND study (Edwards EVOQUE Tricuspid Valve Replacement: Investigation of Safety and Clinical Efficacy after Replacemnt of Tricuspid Valve with Transcatheter Device), 93.8% of patients underwent TTVR via the right femoral vein, with left femoral access used in only 6.2% of cases, which increased to 10.9% in the TRISCEND II trial.^3^^,^^4^ However, the complex interplay between anatomical structures within the tricuspid space, including the inferior vena cava (IVC), RA, TVA, and right ventricle (RV), can make the right-side TF access procedure technically challenging and, in some cases, not feasible.

The EVOQUE TTVR procedure requires a precise delivery system trajectory and an appropriate capsule marker band depth measured from the TVA to successfully deploy the valve. The delivery system trajectory needs to be perpendicular to the native leaflet coaptation, while the capsule marker on the catheter needs to be in between the systolic and diastolic leaflet excursion. This alignment facilitates a safe anchor flip of the EVOQUE valve and helps prevent entanglement with the subvalvular apparatus, device tilting, and device embolization. The trajectory of the delivery system is largely determined by the offset between the IVC and the TVA, whereas the depth of the capsule marker band is primarily influenced by the height of the RA relative to the center of the TVA. Both factors contribute to common technical challenges encountered during TTVR using the currently available EVOQUE system as the current delivery catheter lacks the ability to shed height and only able to add ventricular depth. Maneuvers to gain height, such as pushing the apical guidewire or rotating the delivery catheter, can occasionally be used; however, they carry the risk of right ventricular perforation or altering the catheter trajectory, making the target valve deployment more challenging.

Preprocedural cardiac computed tomography angiography (CCTA) plays a critical role in evaluating the anatomical “working space” for device delivery by assessing the inferior vena cava - tricuspid valve annulus (IVC–TVA) offset and RA height. These measurements help anticipate the degree of flexion required and determine whether sufficient height and depth are available for safe and effective valve deployment. In this report, we describe real-world anatomical scenarios in which a right femoral venous approach may be suboptimal and where an initial left femoral venous approach may be more favorable to overcome specific anatomical constraints.

## Inadequate Right Atrium Height

Among the anatomical factors that influence procedural success, RA height plays a particularly important role in determining the trajectory and depth of the delivery system.

**Figure 1:**
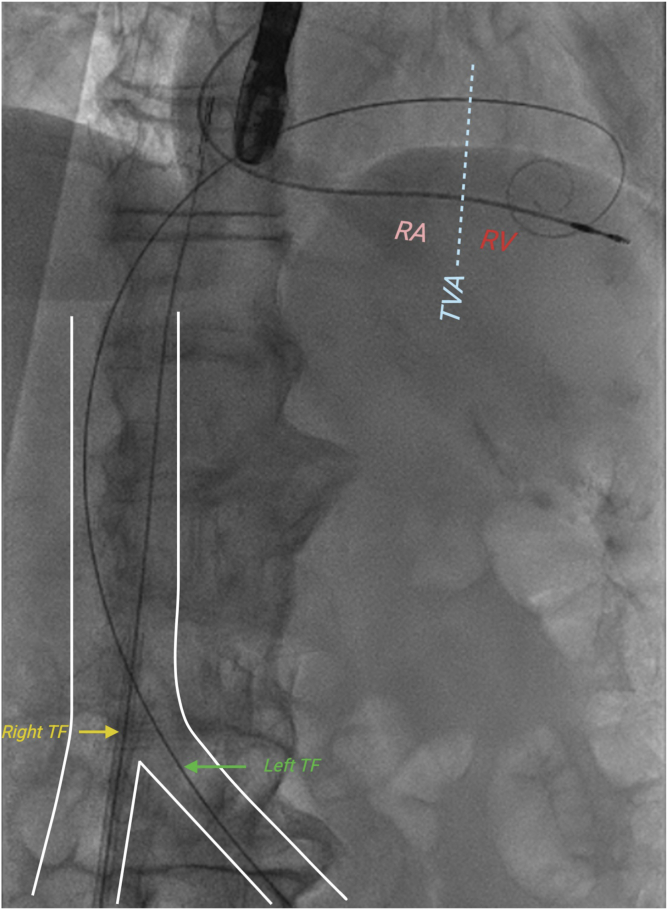
A fair comparison of left- and right-sided femoral venous access for TTVR, highlighting how the left-sided approach follows the outer curvature of the IVC, translating to increased height and improved maneuverability within the RA. Abbreviations: IVC, inferior vena cava; RA, right atrium; RV, right ventricle; TF, transfemoral; TTVR, transcatheter tricuspid valve replacement; TVA, tricuspid valve annulus.

**Figure 2:**
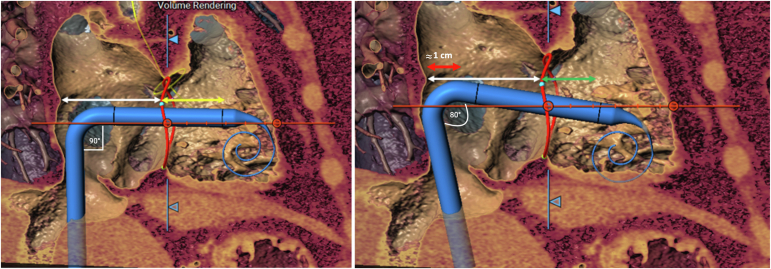
Left image: The white arrow indicates the small RA height, while the yellow arrow shows the capsule gap deeper into the right ventricle as seen with right-sided access. Right image: With left-sided access, the white arrow shows the same RA height, but with an additional 1 cm in right atrial depth (red arrow), while the green arrow demonstrates a more atrial position of the capsule gap. The increase in the catheter primary flex can also be appreciated. Abbreviation: RA, right atrium.

**Figure 3:**
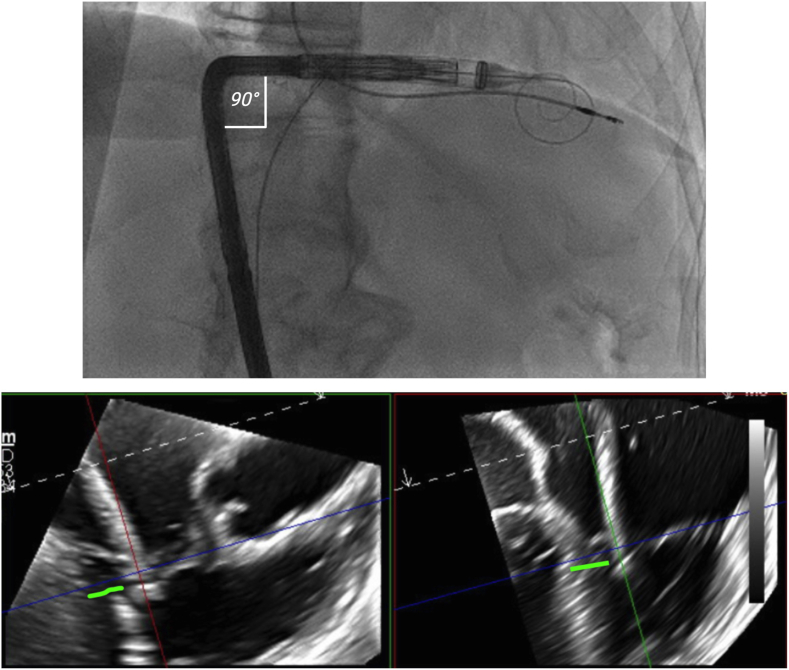
Intraprocedure fluoroscopy and TEE image of the EVOQUE catheter showing closely matching the CT overlay in Figure 2 highlighting the height gain while increasing the need for primary flex on the catheter. The green line indicates the capsule gap that positioned in between the systolic and diastolic leaflet excursion. Abbreviation: TEE, transesophageal echocardiography.

On EVOQUE TTVR preprocedural CCTA planning, RA height is preferably measured in the long-axis view during systole, extending from the midpoint of the TVA to the RA roof, where TVA is defined as the plane of the virtual circumferential ring connecting the basal attachment points of the tricuspid valve leaflets.^5^ In ideal anatomies, a systolic RA height between 60 and 100 mm is generally considered favorable, allowing the catheter to achieve favorable depth. Conversely, in cases with a low RA height (<60 mm), right femoral access may not provide sufficient anterior working room, potentially resulting in excessive RV implantation depth, entanglement with the subvalvular apparatus, a suboptimal trajectory, and noncoaxial alignment of the delivery system with the TVA. Based on device engineering insights, left-sided access typically provides approximately 1 cm of additional RA height compared to right-sided access and biases the catheter more laterally, thereby enhancing RA height and coaxial alignment with the TVA; this was demonstrated through catheter trajectory simulations on benchtop models and further validated by postprocedural imaging assessment. Therefore, left-sided access positions the delivery system along the outer wall of the IVC, creating a more favorable trajectory into the RA position the system higher in the RA and farther from the septum to maximize device height and maneuverability.^6^ Figure 1 shows a fair comparison of left- and right-sided femoral venous access for TTVR, highlighting how the left-sided approach follows the outer curvature of the IVC, translating to increased height and improved maneuverability within the RA. Consequently, Figure 2 illustrates an applied preprocedural simulation of the EVOQUE delivery system, demonstrating depth differences between left and right TF access for TTVR, with the capsule gap extending deeper into the RV from the right side, while appearing more atrial with left-sided access. Furthermore, Figure 3 presents the corresponding intraprocedural fluoroscopy and transesophageal echocardiography images, which show the EVOQUE catheter closely matching the CT overlay, highlighting the height gain while increasing the need for primary flex on the catheter.

## Inferior Vena Cava–Tricuspid Valve Offset

**Figure 4:**
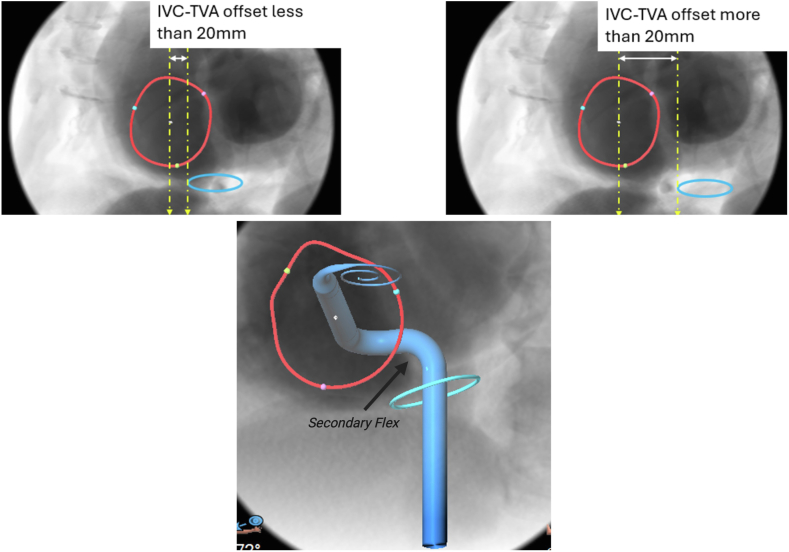
CT studies simulating the IVC-TVA offset at 20 mm showing the need for maximum secondary flex on the catheter for getting the appropriate trajectory. Abbreviations: IVC, inferior vena cava; IVC-TVA offset, inferior vena cava-tricuspid valve annulus offset; TVA, tricuspid valve annulus.

Understanding the relationship between the IVC and TVA is important for explaining why coaxial alignment with the TVA can be particularly challenging in certain anatomies. Accounting for an individual patient's anatomy within the context of the capabilities of the EVOQUE TTVR delivery system is critical for procedural success. In tricuspid intervention, the short-axis (SAX) offset is determined by the location and angle of IVC entry to the RA, which is highly variable, especially in the elderly population with severe symptomatic tricuspid regurgitation.^5^ The IVC-TVA offset, on CCTA, described in Figure 4, is defined in the SAX view as the distance from the near wall of the IVC orifice (blue circle) to midpoint of the TVA (red circle), where the IVC orifice is identified as a horizontal structure on the axial plane at the level of the IVC to RA junction. Further details on the step-by-step approach to quantitatively assess the IVC-TVA relationship have been described previously.^7^

A favorable IVC-TVA relationship is defined as IVC-TVA offset <20 mm. This threshold reflects the catheter’s bridge length, where an offset greater than 20 mm exceeds the catheter’s ability to maintain coaxial alignment with the TVA. In patients with a favorable IVC-TVA relationship, a simple anterior-posterior primary flexion of the delivery system is typically sufficient to achieve coaxial alignment with the TVA. However, a large SAX offset and entry angle would require secondary flexion to direct the delivery catheter more centrally, followed by primary flexion of the TTVR delivery system to direct the catheter towards the TVA.

In patients with a large SAX offset (greater than 20 mm), right femoral access may lead to a nonperpendicular trajectory toward the TVA despite the use of secondary flexion, due to an extremely acute long-axis angle between the mid-IVC and the TVA plane. One strategy to overcome this challenge is to modify the trajectory into the RA by using left TF venous access. Figure 4 demonstrates an IVC–TVA offset greater than 20 mm, highlighting the need for maximal secondary flexion of the catheter to achieve an optimal trajectory toward the TVA.

## Other Considerations in Left-Sided Transfemoral EVOQUE TTVR

**Figure 5:**
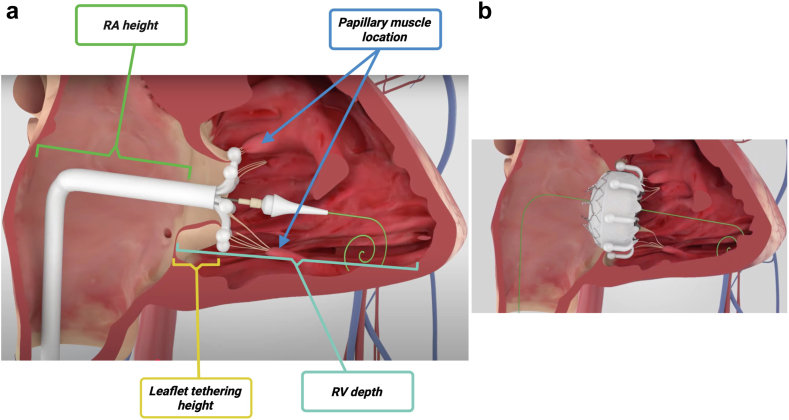
(a) Illustration highlighting key anatomical factors to consider when considering left TF access for TTVR, including RA height, leaflet tethering height, RV depth, and the location of papillary muscles. (b) Image demonstrating optimal valve deployment with excellent coaxial alignment and successful leaflet capture by all anchors. Reproduced with permission from Edwards Lifesciences. Abbreviations: RA, right atrium; RV, right ventricle; TF, transfemoral; TTVR, transcatheter tricuspid valve replacement.

The feasibility of a left-sided access route for TTVR is not determined by a single parameter, such as RA height or IVC–TVA offset, but rather by a comprehensive assessment of multiple anatomical factors. These include RA height, leaflet tethering height (for adequate leaflet capture), RV depth, and the location of papillary muscles (potentially affecting risk of interaction with subvalvular structures) Figure 5. This interplay ultimately determines the safety and suitability of the left TF approach.

An important consideration is weighing the advantages and disadvantages of initially attempting right TF access before switching to a left-sided approach, versus preceding directly with left-sided TF access. A potential disadvantage of the stepwise approach is the theoretical risk of increased vascular or bleeding complications, infection, and prolonged procedure time due to the need for 2 large-bore femoral accesses in a single procedure. Moreover, if switching from right to left TF access, the same EVOQUE device can typically be reused, provided that the capsule edge remains uncompromised, which should be carefully checked before reintroduction.

**Figure 6:**
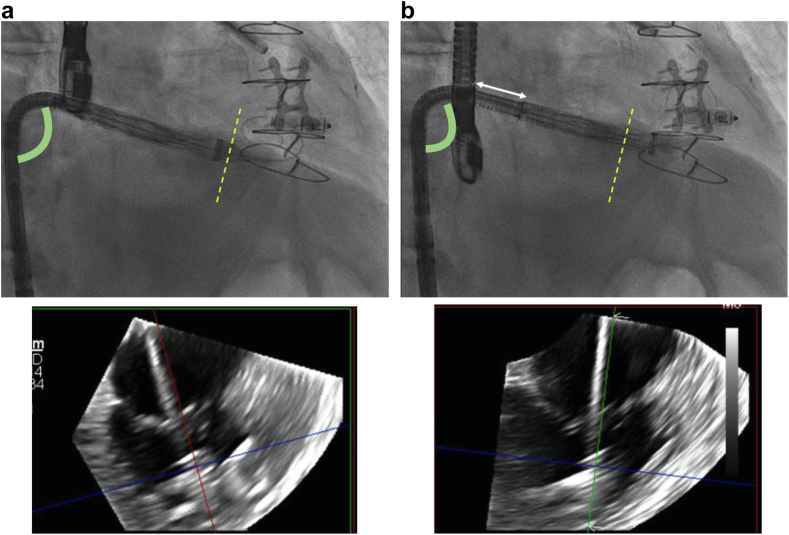
(a) The capsule edge marker is positioned above the native tricuspid annulus (dotted yellow line), reflecting a more atrial orientation of the delivery system due to left-sided access. (b) Additional depth (white arrow) brings the valve below the annulus. The primary flex angle (green angle), exceeding 90 degrees, is also demonstrated.

In certain scenarios, the right femoral vein may be inaccessible for percutaneous access due to factors such as excessive scarring, complications from prior interventions (e.g., right femoral arteriovenous fistula), or extrinsic factors like orthopedic hardware, thereby necessitating the use of a left TF approach for safe large-bore access. However, this can introduce challenges of its own, such as excessive and undesired RA height, potentially increasing procedural complexity by making it more difficult to achieve adequate depth without compromising the trajectory and control of the delivery system. Figure 6 demonstrates the impact of left-sided access, showing increased device height with the capsule edge marker positioned above the native TVA due to the delivery system catheter staying more atrial, and the subsequent need for added depth and increased primary flex.

In addition, the left TF approach for TTVR can present other challenges due to the tortuous anatomy of the left femoral vein and the acute angulation at the junction where the common iliac veins merge into the IVC, which may hinder advancement of the delivery system. In such cases, aggressive dilation using a long 33-French dilator can facilitate successful advancement of the delivery system. Maintaining the dilator in place for approximately 30 seconds can help straighten the venous pathway and reduce resistance, thereby improving trackability of the delivery system.

Furthermore, when considering a left-sided approach, it is important to assess for potential compression of the left common femoral vein by the overlying artery, which is an anatomic consideration that may prevent catheter advancement. This should be carefully evaluated on preprocedural CCTA.

With increasing real-world use of TTVR, it is likely that patients with a broader range of right-sided anatomies will need treatment. In cases where TF access, whether right or left, is not feasible, IJ access may be considered as an alternative. A single-center study demonstrated that the right TJ with EVOQUE can be a safe, feasible, and effective alternative vascular access site.^8^ However, TJ access introduces distinct logistical and procedural challenges, such as altered catheter orientation, limited operator experience, nonstandard operator positioning, and increased radiation exposure. Moreover, navigating large-bore delivery systems through the IJ vein presents unique challenges in sheath control, as well as risk of injury to nearby anatomical structures.

## Conclusion

Variability in right heart anatomy can impact the ability of current TTVR devices to achieve coaxial alignment with the TVA. In select real-world scenarios, such as low RA height (less than 60 mm) or a large IVC–TVA offset which is an offset distance greater than 20 mm, left TF access may serve as a safe and effective approach when anatomical constraints limit the feasibility of right-sided access. Preprocedural CCTA plays a critical role by identifying the optimal approach to minimize technical challenges. Future studies will show the feasibility and safety of left TF access of EVOQUE TTVR and further characterize anatomic variability and support the development and design of next-generation delivery systems iterations capable of safely accommodating a wider range of anatomies.

## Funding

The authors report no funding relevant to the contents of this paper to disclose.

## CRediT Authorship Contributions

Antonio H. Frangieh contributed to the conception of the manuscript, provided critical revisions for important intellectual content, and supervised the overall project. Mark W. Abdelnour drafted the manuscript and created the figures. Siddharth Vad provided critical insights for important intellectual content. Scott Chadderdon revised the manuscript critically for important intellectual content. Jin Kyung Kim edited the manuscript and provided appropriate revisions. Firas Zahr contributed to the conception of the manuscript, critically revised the manuscript, and provided important edits and comments.

## Disclosure Statement

A.H. Frangieh is a consultant and received institutional research grant from Edwards Lifesciences. F. Zahr and S. Chadderdon are consultants and receive research and educational grants from Edwards Lifesciences, Philips, and Medtronic. The other authors had no conflicts to declare.
